# Intraoperative transfusion practice in burned children in a university hospital over four years: a retrospective analysis

**DOI:** 10.1186/s12871-021-01336-3

**Published:** 2021-04-15

**Authors:** Eva Wittenmeier, Astor Katharina, Irene Schmidtmann, Eva-Verena Griemert, Marc Kriege, Tatjana König, Pirlich Nina

**Affiliations:** 1 Department of Anesthesiology, University Medical Centre of Johannes Gutenberg University, Langenbeckstraße 1, 55131 Mainz, Germany; 2 Department of Anesthesiology and Intensive Care, Catholic Clinical Centre, Mainz, Germany; 3 Institute of Medical Biostatistics, Epidemiology and Informatics, University Medical Centre of Johannes Gutenberg University, Mainz, Germany; 4 Department of Pediatric Surgery, University Medical Centre of Johannes Gutenberg University, Mainz, Germany

**Keywords:** Blood transfusion, Transfusion thresholds, Pediatric burn injury, Patient blood management, Pediatric, Red blood cells

## Abstract

**Background:**

Patient blood management programs should be applied to the pediatric population, but little is known about the current transfusion practice of pediatric burn injury patients. This retrospective study was performed to evaluate the practice of red blood cell (RBC) transfusion in children with burn injury, their predictive factors, and adherence to the German transfusion guideline.

**Methods:**

We reviewed the RBC transfusion practice of all children younger than 8 years with burn injury who were operated during a four-year period in a German university medical center. We analyzed the data associated with transfusion and guideline conformity of transfusion triggers for RBCs from the beginning to the end of hospital stay using logistic regression.

**Results:**

During the four-year period, 138 children (median age 21 months, minimum-maximum 9–101 months) with burn injury needed surgery, 31 children were transfused with RBCs. During their hospital stay, the median hemoglobin concentrations (Hb) of transfused and non-transfused children were 8 g/dL (6.3–11.3 g/dL) and 10.7 (7–13.8 g/dL), respectively. Total body surface area burned (TBSA) (OR = 1.17 per % TBSA, 95% CI = [1.05; 1.30], *p* = 0.0056), length of surgery (OR = 1.016 per minute, 95% CI = [1.003; 1.028], *p* = 0.0150), and Hb (OR = 0.48 per 1 g/dl in Hb, 95% CI = [0.24; 0.95], *p* = 0.0343) were associated with transfusion while other factors (age, gender, ASA, and catecholamines) did not show notable association. Length of stay was mainly influenced by TSBA (+ 1.38 days per %, *p* <  0.0001), age (+ 0.21 days per month, *p* = 0.0206), and administering of catecholamines (+ 14.3 days, *p* = 0.0118), but not by RBC transfusion. The decision to transfuse was in 23% too restrictive and in 74% too liberal according to the German guidelines.

**Conclusions:**

Amount of TBSA, length of surgery, and Hb influenced the RBC transfusion rate in burned children. However, age and length of stay were not affected by transfusion of RBCs. In clinical practice of burned children, physicians follow a more liberal transfusion strategy than the proposed in guidelines.

**Supplementary Information:**

The online version contains supplementary material available at 10.1186/s12871-021-01336-3.

## Background

Red blood cell (RBC) transfusion might be life-saving in severe cases of anemia in perioperative and critical care settings. However, in recent years, adverse effects of blood transfusion on postoperative morbidity and mortality have been observed [1–4]. In consequence, patient blood management (PBM) programs have been developed in order to optimize the utilization of blood components and thereby improve the clinical outcome and patient safety. After prospective clinical trials showed that it is safe to start transfusion at a lower hemoglobin (Hb) threshold, restrictive transfusion practice was recommended with caution at higher risk of oxygen supply [5, 6]. Current research focuses on establishing the adequate transfusion rules for patients. Current transfusion practice is not known for pediatric population, but surveys and clinical studies suggest that striking variability exists in perioperative pediatric RBC transfusion practice [7–9].

Reports of Serious Hazards of Transfusion (SHOT) show that the incidence of adverse events is higher in infants. In addition, in the recent SHOT report pediatric cases continue to be over-represented in several categories, particularly in under- and over-transfusion and in incorrect blood component transfusion [10]. Furthermore, children are supposed to be at higher risk for noninfectious adverse events like febrile nonhemolytic transfusion reaction, TACO (transfusion associated circulatory overload) and TRALI (transfusion related acute lung injury). Mortality associated with noninfectious risks is higher than in adults [11, 12]. Few blood transfusion guidelines address blood transfusion in children and recommend a restrictive approach [13–16]. Pediatric patients with burn injury often require RBC transfusion during their hospital stay. As burned pediatric patients are immunosuppressed and RBC transfusion may affect the immune system [17], a restrictive approach in particular might prove beneficial for them [18]. In adults with burn injury, data from a prospective study suggest that the outcome of both the restrictive and liberal blood transfusion strategy is the same [19]. There is a lack of prospective studies on burned pediatric patients. Cohort studies examining the change of transfusion practice in burned children suggest that a restrictive transfusion policy does not have any adverse effect on patient outcome and may prove cost-effective. However, little is known about the current transfusion practice of pediatric population and their adherence to current guidelines.

## Methods

This retrospective study was performed to evaluate the transfusion practice in children with burn injury, their predictive factors, and adherence to the German guidelines for blood transfusion. For this purpose, we reviewed all children younger than 8 years and who underwent surgery for burn injury from January 1, 2010 to January 1, 2015. The local ethics committee (Rhineland Palatine, Germany) had approved this study. Pre-, intra-, and postoperative transfusion data and other perioperative care data of these children were identified by screening their operation schedule, and subsequently, data were obtained by revising the children’s patient charts. Transfusion, laboratory, application of catecholamines and other patient data were collected from the beginning to the end of hospital stay. Additionally, postoperative length of stay in intensive care unit (ICU) and overall length of stay in hospital were evaluated. Children with incomplete or lack of key information of their anesthesia protocol were excluded. The hemoglobin concentration (Hb) before the first transfusion of RBC was compared with the proposed Hb triggers of the German transfusion guidelines for children older than 4 months [16] (Table 1). Transfusion thresholds have not changed with regard to Hb as per the German transfusion guideline during our study period and are still valid today.

**Table 1:** Hemoglobin thresholds of the German guideline of blood transfusion for children

Patient criteria	Lowest acceptable hemoglobin threshold according to the German guidelines of blood transfusion
> 4 months hemodynamically stable	**6 g/dL**
> 4 months hemodynamically instable	**10 g/dL**

For this purpose, children who had transfusion were classified into two groups: Hb value of children that needed catecholamines directly before or at the start of transfusion was compared with the Hb threshold for instable children (10 g/dL). Hb values of transfused children that did not need catecholamines were compared with the threshold for stable children (6 g/dL).

For categorical variables, absolute and relative frequencies were provided; median, minimum, and maximum were calculated. Potential influence of the explanatory covariates on the decision to transfuse was assessed by using logistic regression. We used multiple imputation (MI) for sensitivity analysis as potential explanatory variables for all patients had not been documented. MI was performed by SAS PROC MI, and the results were combined by SAS PROC MIANALYZE. We performed 10 imputations, and in order to impute missing values, we used transfusion, age, gender, operation procedure, American Society of Anesthesiologists’ (ASA) classification, height, weight, duration of surgery, minimal Hb, Quick, lactate, proportion of TBSA, postoperative length of stay, and length of stay in ICU. We used covariance analysis to assess the effect of transfusion on length of stay, adjusting for age, gender, minimal Hb, proportion of TBSA, ASA, and operation procedure. Again, we performed both complete case (CC) and MI analyses. The results of the CC were presented in the main paper, and the results of the MI were presented in supplementary materials.

Differences were noted in the circulatory parameters before and after transfusion using the t-test and Wilcoxon test. All tests were performed with exploratory intention; hence, *p* values are descriptive in nature. Nevertheless, p values < 0.05 were considered statistically significant..

## Results

**Fig. 1:**
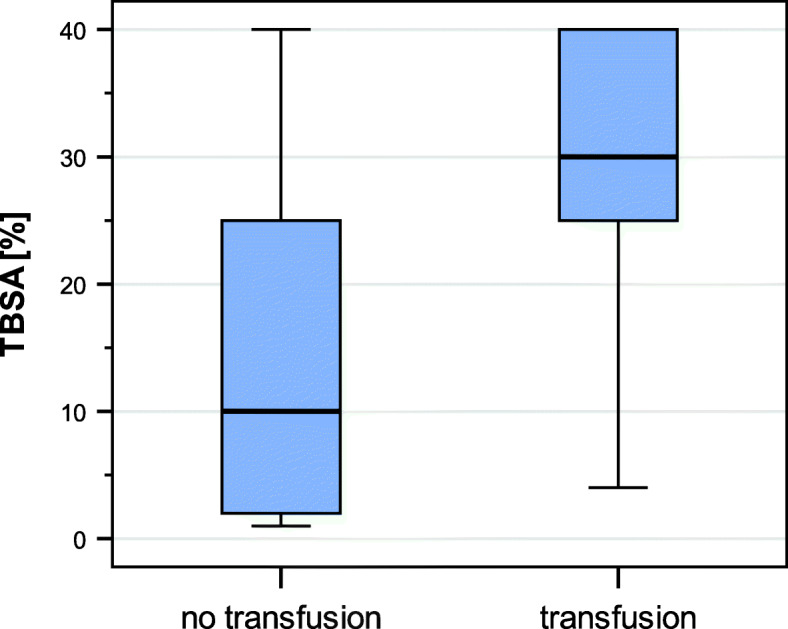
Total body surface area burned (TBSA) influences the probability of transfusion with red blood cells. Children with burn injury without transfusion of RBC had a median TBSA of 10% (minimum-maximum 1–40) and children with transfusion had 30% (4–40) (*p* < 0.0001)

During the four-year period, a total of 152 patients were eligible for the study; however, 14 patients’ charts were incomplete or with insufficient key information, thus 138 patients were analyzed. Of these 31 were transfused with RBCs, 18 of them additionally with fresh frozen plasma. One patient was only transfused postoperatively, whereas another patient’s transfusion has started preoperatively and was going on intraoperatively, and the rest of patients were transfused intraoperatively. Cell saver was not used at all, and fibrinogen was given in 9 transfused and 3 non transfused children. Patient characteristics are shown in Table 2. Children without RBC transfusion had a median area of 10% TBSA, whereas children with transfusion had a median area of 30% TBSA (Table 2 and Fig. 1).

**Table 2:** Characteristics of children and surgery

Patient characteristics	All children ***n*** = 138	Children with transfusion of RBC ***n*** = 31	Children without transfusion of RBC ***n*** = 107	***p***-value*
Gender (years) *N* = 138	m: 83 (60%) f: 55 (40%)	m: 23 (74%) f: 8 (26%)	m: 60 (56%) f: 47 (44%)	0.0952
Age (months) *N* = 138	21 (9–101)	19 (12–93)	21 (9–101)	0.5568
Weight (kg) *n* = 136 (31/105)^**^	12 (7–30)	12 (9.5–30)	12 (7–30)	0.7463
ASA Classification *N* = 130 (29/101)	1: 34 (26%)	1: 4 (14%)	1: 30 (30%)	0.0155
	2: 72 (55%)	2: 14 (48%)	2: 58 (57%)	
	3: 16 (12. %)	3: 7 (24%)	3: 9 (9%)	
	4: 8 (6%)	4: 4 (14%)	4: 4 (4%)	
Prothrombin time *n* = 44 (17/27)	97% (40–125%)	91% (40–124%)	100% (64–125%)	0.1656
Lactate level *n* = 47 (28/19)	1.0 (0.3–4.8) g/dL	1.0 (0.3–4.8) g/dL	1.0 (0.3–2.8) g/dL	0.8708
Proportion of TBSA	15% (1–40)	30% (4–40)	10% (1–40)	< 0.0001
Length of surgery (min) *N* = 138	72 (5–475)	210 (28–475)	47 (5–400)	< 0.0001
Minimal hemoglobin (g/dL) *N* = 91 (31/60)	9.6 (6.3–13.8)	8 (6.3–11.3)	10.7 (7.0–13.8)	< 0.0001
Postoperative length of hospital stay (days) n = 138	11 (1–94)	41 (9–94)	8 (1–91)	< 0.001
catecholamines N = 138	Yes: 14 (10%) No: 124 (90%)	Yes: 8 (26%)^a^ No: 23 (74%)	Yes: 6 (6%)^b^ No: 101 (94%)	0.0032

*TBSA* total burned body surface area, *ASA* American Society of Anesthesiologists, *m* male, *f* female. Values are absolute numbers (proportion) or median (minimum-maximum)

^a^applied directly before or while transfusion ^b^applied during the hospital stay

*p values for comparison between children with and without transfusion

**Numbers in brackets give numbers of children with and without transfusion for whom measurement is available

In the CC logistic regression analysis, we found that minimal Hb (OR = 0.48 per 1 g/dl in Hb, 95% CI = [0.24; 0.95], *p* = 0.0343), length of surgery (OR = 1.016 per minute, 95% CI = [1.003; 1.028], *p* = 0.0150), and TBSA (OR = 1.17 per % TBSA, 95% CI = [1.05; 1.30], *p* = 0.0056 were associated with transfusion. Age, sex, ASA, and catecholamines did not show any association with transfusion (Table 3).

**Table 3:** Association of explanatory factors on transfusion, complete case analysis

	Odds Ratio		
	Point estimate	95% Confidence interval	p
Age (per year)	0.99	[0.95; 1.03]	0.6389
Gender (female vs male)	0.45	[0.03; 5.94]	0.5445
TBSA (per %)	1.17	[1.05; 1.30]	**0.0056**
ASA: 2 vs 1	0.37	[0.02; 6.34]	0.8162
ASA: 3 vs 1	0.11	[0.003; 5.09]	0.2692
ASA: 4 vs 1	0.25	[0.01; 8.86]	0.7697
Length of surgery (per minute)	1.016	[1.003; 1.028]	**0.0150**
Minimal hemoglobin (per g/dL)	0.48	[0.24; 0.95]	**0.0343**
Catecholamines	1.27	[0.15; 10.53]	0.8243
Necrectomy	0.82	[0.03; 22.35]	0.9502
Skin harvesting and grafting	1.33	[0.15; 11.98]	0.8011
Change of dressings	1.02	[0.04; 27.19]	0.9919
Wound refreshment	1.33	[0.06; 31.28]	0.8614

The outcome of the logistic regression analysis for predicting transfusion based on MI is similar to the CC analysis. Minimal Hb (OR = 0.48, 95% CI = [0.26; 0.90], *p* = 0.0230), length of surgery (OR = 1.017 per minute, 95% CI = [1.005; 1.029], *p* = 0.0063), and TBSA (OR = 1.15 per % TBSA, 95% CI = [1.04; 1.28], *p* = 0.0069) are associated with the transfusion, while the other covariates do not exhibit notable association with transfusion (an Additional file 1 shows this in more detail in Table 3b).

Neither CC nor MI found an association of transfusion and length of stay after adjusting for other variables, which are likely to be associated with length of stay. Length of stay was mainly influenced by TSBA (+ 1.38 days per % ([CC]), *p* <  0.0001; + 1.08 days per % ([MI]), *p* <  0.0001), age (+ 0.21 days per month ([CC]), *p* = 0.0206; + 0.19 days per month ([MI]), *p* = 0.0026), and administering of catecholamines (+ 14.3 days ([CC]), *p* = 0.0118; + 13.24 ([MI]), *p* = 0.0080). Necrectomy influenced length of stay in the MI (+ 14 days, *p* = 0.0146), but not in the CC (+ 8.43 days, *p* = 0.2353).

**Fig. 2:**
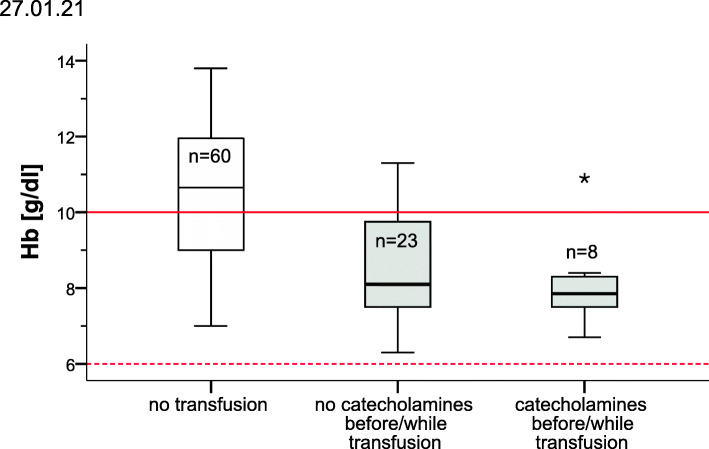
Practice of transfusion compared to the German guidelines of blood transfusion. The examined hemoglobin concentrations (Hbs) are the minimal Hbs during hospital stay (for children who did no receive red blood cells [RBCs]) or the measured Hbs before transfusion of RBCs (for children who received transfusion). The Hb-threshold lines (red) represent the Hb-thresholds of the German guideline of blood transfusion for stable (6 g/dl) or instable (10 g/dl) children > 4 months [16]. The diagram shows that all hemodynamically stable children were transfused at hemoglobin triggers higher than recommended in the German guidelines. Transfused children that received catecholamines were transfused too restrictive according to the German guidelines. Overall the decision to transfuse was too liberally in 74, too restrictive in 23% and in 3% adequate according to the German guidelines

Hemoglobin concentration was measured in 91 children (31 transfused/60 non-transfused) and compared with the German transfusion guidelines (Table 2 and Fig. 2). The median minimal hemoglobin concentration of non-transfused children was 10.7 g/dL and that of transfused children was 8.0 g/dL. Hb was collected from every child before transfusion, but not controlled in five children after transfusion. The decision to transfuse was too restrictive in 7/31 (23%, all of them were children with catecholamines), too liberal in 23/31 (74%), and correctly in one case (3%) according to the German guidelines. The median increase of hemoglobin was 1.3 g/dL; the median transfused volume of RBC was 13,6 ml/kg (4.2–66.7 min-max). Transfusion of RBC had a measurable effect on circulatory parameters like Hb, heart rate, and SAP (Table 4).

**Table 4:** Effects of red blood cell transfusion on circulatory parameters in children with burn injury

Measured parameters	Before transfusion	After transfusion	p-value
Hemoglobin concentration	8.0 g/dL (6.3–11.3) n = 31	9.8 g/dL (7.8–13.0) *n* = 26	p < 0.001
Increase of hemoglobin after transfusion		1.3 (−1.1–5.0) *n* = 26	
Heart rate	125/min (90–160)	120/min 85–155)	*p* = 0.017
Systolic blood pressure	80 mmHg (60–125)	93 mmHg (80–115)	*p* < 0.001

Values are median (minimum-maximum)

## Discussion

In our study, TBSA, length of surgery, and Hb influenced the transfusion rate in burned children. Length of stay was not influenced by RBC transfusion. According to the German transfusion guidelines, more than half of the children were transfused too liberally.

Non-transfused children had a median area of 10% TBSA, whereas transfused children had a median area of 30% TBSA. A multicenter retrospective study in a mixed population of adults and children with burn injury found that 75% of patients with TBSA > 20% received blood transfusion [20]. Another observational study evaluating predictive factors for blood transfusion in adults and children found TBSA > 20% is a useful predictor for blood transfusion [21]. Only few data are available to evaluate the association between TBSA and transfusion in an exclusively pediatric collective study like ours. Interestingly, another retrospective study found in transfused children with burn injury a mean TBSA of 29 to 31%. This corresponds to our findings. Information of TBSA of non-transfused children were not evaluated in this study [22]. Another retrospective cohort study evaluated the effects of a change to a restrictive blood transfusion protocol in acute pediatric burn injuries. Children who received blood had a mean TBSA of 45% (before the protocol change) and 43% (after the protocol change), respectively. TBSA of children who did not receive blood transfusion was not described [23].

In our study, the length of surgery also correlated with the transfusion rate. Obviously, this can be explained by the fact that surgeons need more time for larger TBSA.

Age did not influence transfusion rate. This statement contrasts the other finding that suggests younger age is a risk factor for transfusion. In an observational study examining transfusion practice in children older than 28 days who underwent surgery in different hospitals was found that younger age (29 days to 2 years) among other factors was a preoperative variable associated with increased odds of having an intraoperative or postoperative RBC transfusion [7]. A recently published register study in pediatric patients undergoing complex cranial vault reconstruction identified that age less than 24 months as one of the factors for increased RBC transfusion [24].

Length of stay was mainly influenced by whether it was an emergency procedure, TSBA, and age, not by the transfusion rate. Prospective studies examining this subject in children are rare. Lacroix et al. compared in a prospective, multicenter study in stable, critically ill children in ICU restrictive with liberal RBC transfusion strategies and found no difference in length of stay in ICU [25]. Retrospective studies in burned children with protocol changes showed that days of hospitalization were less [23] or the same [22] after the change. Prospective data investigating the impact of transfusion on length of total hospital stay in this special population are to the best of our knowledge not available.

In our study, transfusion had a measurable effect on heart rate and systolic blood pressure; however, the effect is small, thus we cannot exclude that this effect is due to other factors such as catecholamines.

Our study evaluated the minimal hemoglobin concentration of transfused and non-transfused children during their hospital stay. Lower hemoglobin concentration was associated with RBC transfusion. The median minimal hemoglobin concentration of non-transfused children was 10.7 g/dL, and 8 g/dL before receiving RBC. The German guidelines for blood transfusion recommend a threshold of 6 to 7 g/dL (hemodynamically stable) and 10 g/dL (hemodynamically unstable) for children older than 4 months with active bleeding [16]. These guidelines do not specifically address children with burn injury, thus special Hb thresholds for this collective are not available in a guideline because available prospective randomized data of critically ill children without acute blood loss cannot be fully be applied to pediatric patients with burn injury [26] International guidelines propose lower guidelines for children with bleeding, but they do not specify Hb thresholds for unstable children because of insufficient data [13, 14]. 74% of the children were transfused too liberally. Most of the too liberal transfusions happened in children that did not receive catecholamines. The reason for this could be the threshold of 6 g/dL for stable children is a very restrictive approach that is not yet practiced. In a British multicenter observational study of RBC transfusion practice in children with a median age of 5 years, the median pre-transfusion hemoglobin was 7.9 g/dL [8]. On the other hand, the small increase of Hb after transfusion in our study is a restrictive approach that corresponds to the recommendation that post-transfusion Hb should not be > 2 g/dl above the transfusion threshold [27]. Unfortunately, database for risks or benefits of a restrictive transfusion approach versus a liberal one in pediatric patients is very small. One prospective landmark study of Lacroix et al. showed that restrictive transfusion strategy in stable critically ill children does not increase adverse events [25]. A few clinical studies have examined blood transfusion effects retrospectively [28–30]. Similar to adults, they showed increased incidence of 30 days mortality, postoperative infections, and correlation between the volume of RBC transfused and the incidences of adverse outcomes [30], length of mechanical ventilation, and length of ICU stay [28, 29].

For the subgroup of children with burn injury a recent study showed that restrictive blood transfusion with a hemoglobin threshold of 7 g/dL protocol in acute pediatric burn care is safe, may reduce medical risks, and lower economic burden [23]. Transfusion-related immunomodulation (TRIM) is one of the effects of allogeneic blood transfusion. To what extent these immunomodulatory effects alter clinical outcomes remains controversial [31]. Children with burn injury are immunosuppressed, and massive transfusion could put them at risk for TRIM, transfusion-related graft-versus-host reaction and enhanced allograft survival. However, these effects are yet to be examined in pediatric population. Retrospective studies showed a potentially higher risk for the development of sepsis [18] In a prospective study among adults, wound healing, mortality and infection did not differ between restrictive and liberal transfused adult patients with burn injury [19].

Children with burn injury are immunosuppressed. Therefore, the risk of this complication should be minimized. In a retrospective cohort study, children with burn injury of 60% TBSA and concomitant inhalation injury were more likely to develop sepsis after transfusion of high amounts of blood products [18]. Hence, children with burn injury could benefit from a restrictive transfusion practice in particular. Apart from that restrictive transfusion practice minimizes other adverse effect of transfusion in children like hyperkalemia [26], TACO, febrile hemolytic transfusion reactions and incorrect blood component use [11, 12]. Indeed, prospective studies in burned children examining the effects and safety of restrictive versus liberal regimen are lacking. In summary, the transfusion practice in the examined collective reflects that children are transfused more liberally than possible or recommended. In addition, PBM strategies helping to avoid transfusion were not (cell saver) or poorly (fibrinogen or tranexamic acid (TXA)) applied. Recommendations for the use of TXA, fibrinogen, and cell saver in pediatric patients have been made during the last 10 years [11, 13, 15, 32, 33]; however data whether these strategies are really practiced in pediatric perioperative transfusion practice is insufficient. Therefore, this study suggests a possible gap between recommendation and reality.

## Conclusion

In this retrospective collective, the amount of TBSA, Hb, and length of surgery influenced the transfusion rate. Age and length of stay were not influenced by RBC transfusion. According to the German guidelines of blood transfusion, most of the children were infused too liberally, indicating that restrictive hemoglobin triggers are not yet implemented in clinical practice of pediatric anesthesia in pediatric burn care.

## Supplementary Information

**Additional file 1: Table 3b supplement.** Characteristics of children and surgery using multiple imputation.

## Data Availability

The datasets used and/or analyzed during this study are available from the corresponding author on reasonable request.

## Abbreviations

ASA: American Society of Anesthesiologists
CC: Complete case analysis
Hb: Hemoglobin concentration
LOS: Length of stay
MI: Multiple imputation
OR: Odds ratio
RBC: Red blood cell
SAP: Systolic arterial pressure
TBSA: Total body surface area burned
