# Supplementary material for: Intraoperative transfusion practice in burned children in a university hospital over four years: a retrospective analysis
Source: BMC Anesthesiol. 2021 Apr 15;21:118. doi: 10.1186/s12871-021-01336-3 (PMC8048155; doi:10.1186/s12871-021-01336-3)
Supplement: Supplementary file 1 — Additional file 1: Table 3b supplement. Characteristics of children and surgery using multiple imputation. [file 12871_2021_1336_MOESM1_ESM.docx]

| **Additional file 1 Table 3b supplement**: Characteristics of children and surgery using multiple imputation | | | |
| --- | --- | --- | --- |
|  | **Odds Ratio** | |  |
| Patient characteristics | **Point estimate** | **95% Confidence interval** | **p** |
| Age (per year) | 0.98 | [0.955;1.02] | 0.4054 |
| Gender (female vs male) | 0.61 | [0.06; 6.38] | 0.6805 |
| TBSA (per %) | 1.15 | [1.04; 1.28] | **0.0069** |
| ASA classification: 2 vs 1 | 0.32 | [0.02; 4.95] | 0.4120 |
| ASA classification: 3 vs 1 | 0.12 | [0.003; 4.68] | 0.2556 |
| ASA classification: 4 vs 1 | 0.23 | [0.007; 8.28] | 0.4224 |
| Length of surgery (per minute) | 1.017 | [1.005; 1.029] | **0.0063** |
| Minimal hemoglobin (per g/dL) | 0.48 | [0.26; 0.90] | **0.0230** |
| Catecholamines | 1.58 | [0.18; 13.63] | 0.6755 |
| Necrectomy | 1.86 | [0.10;36.14] | 0.6804 |
| Skin harvesting and grafting | 0.68 | [0.09;5.10] | 0.7081 |
| Change of dressings | 1.21 | [0.06;24.32] | 0.9005 |
| Wound refreshment | 1.17 | [0.05;25.67] | 0.9212 |
| TBSA = total burned body surface area, ASA = American Society of Anesthesiologists, m = male, f = female | | | |
